# Lactate Promotes the Second Cell Fate Decision in Blastocysts by Prompting Primitive Endoderm Formation Through an Intercellular Positive Feedback Loop That Couples Paracrine FGF Signalling

**DOI:** 10.1111/cpr.70088

**Published:** 2025-06-27

**Authors:** Xiao Hu, Yawen Tang, Wei Zhao, Juan Liu, Zhize Liu, Qianyin Yang, Meiqiang Chu, Jianhui Tian, Lei An, Shumin Wang

**Affiliations:** ^1^ Frontiers Science Center for Molecular Design Breeding Of the Ministry of Education, China Agricultural University Beijing China; ^2^ Key Laboratory of Animal Genetics, Breeding and Reproduction Of the Ministry of Agriculture and Rural Affairs, China Agricultural University Beijing China; ^3^ State Key Laboratory of Animal Biotech Breeding China Agricultural University Beijing China; ^4^ National Engineering Laboratory for Animal Breeding China Agricultural University Beijing China; ^5^ College of Animal Science and Technology, China Agricultural University Beijing China; ^6^ Center for Reproductive Medicine Renji Hospital, School of Medicine, Shanghai Jiao Tong University, and Shanghai Key Laboratory for Assisted Reproduction and Reproductive Genetics Shanghai People's Republic of China; ^7^ College of Animal Science and Technology, Hunan Provincial Key Laboratory for Genetic Improvement of Domestic Animal Hunan Agricultural University Changsha China

**Keywords:** FGF4, histone lactylation, lactate, pre‐implantation embryo, primitive endoderm

## Abstract

Lactate has been widely recognised as an energy source and metabolic by‐product, but increasing evidence supports its critical role as a signalling molecule or epigenetic substrate. During early embryogenesis, lactate production increases during the transition from early to late blastocyst, coinciding with the differentiation of inner mass cell (ICM) into epiblast (EPI) and primitive endoderm (PrE), termed the second cell fate decision. However, the role of this hallmark metabolic change in the second cell fate segregation remains unknown. Herein, using in vitro and in vivo models, we found lactate production is preferentially increased in PrE cells and is essential for ICM differentiation into PrE. Mechanically, increased lactate in PrE precursor cells and FGF signalling in EPI precursor cells reciprocally activate each other and synergise to prompt PrE specification, forming an intercellular positive feedback loop essential for this lineage commitment. Additionally, lactate enhanced histone lactylation levels during differentiation into PrE fate. Thus, our findings construct a complex multilayer model in which intracellular metabolite in PrE cooperates with intercellular growth factor signalling from EPI to regulate early embryonic lineage commitment. Highlighting the multifaceted lactate's function, our findings also advance the current knowledge that bridges epigenetic reprogramming and metabolic remodelling during early embryonic development.

## Graphical abstract

Paracrine FGF4 from EPI precursor cells promotes lactate synthesis within PrE precursor cells, while the increased lactate in turn stimulates FGF‐ERK signalling via lactate shuttling. The increased lactate in PrE precursor cells directly promotes PrE formation by elevating the level of histone lactylation.

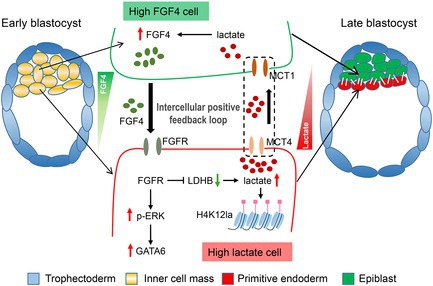

## 1 Introduction

The formation and development of blastocysts is a landmark event during mammalian embryogenesis. During this process, two sequential cell fate decisions occur to form three distinct cell types [1, 2]. At the early blastocyst stage, embryos complete the first cell fate decision to form the inner cell mass (ICM) and trophectoderm (TE). Subsequently, from early blastocyst to late blastocyst stage, ICM cells separate to epiblast (EPI), which gives rise to the embryo proper, or the extra‐embryonic primitive endoderm (PrE), which later forms the parietal endoderm (PE) and visceral endoderm (VE) [1, 3, 4]. Moreover, the genetic ablation of either GATA binding protein 6 (*Gata6*) or fibroblast growth factor receptor 1 (*Fgfr1*) disrupts the establishment of PrE and finally leads to embryonic lethality during or shortly after implantation [5, 6], highlighting the importance of proper differentiation of PrE for successful mammalian embryonic development. Over decades, a variety of studies have demonstrated that differentiation of the EPI and PrE is orchestrated through intricate interactions centred around the NANOG‐GATA6‐FGF/ERK regulatory network [7, 8, 9, 10, 11, 12]. Primarily, paracrine FGF4 from EPI acts on adjacent cells to modulate the expression of *Nanog*, *Gata6* and *Gata4*, thereby inducing a PrE fate [8, 9, 13].

Intriguingly, in addition to the activity of prominent FGF‐ERK signalling axis among different lineages, dramatic changes in metabolism coincide spatiotemporally with cell fate commitment during this process [13, 14, 15, 16, 17]. However, despite the importance of metabolic control in pluripotency acquisition [18, 19, 20], cell fate decisions [21, 22, 23], even in the separation of TE and ICM [16, 24], the role of metabolic variation in determining the second cell fate remains largely unknown. Specifically, a dramatic energy metabolism remodelling occurs in the ICM during the second cell fate decision, as revealed by a decrease in oxidative phosphorylation activity, as well as a concurrent sharp increase in glycolytic activity and lactate synthesis [14, 16, 25]. Lactate has been discovered as signalling molecule, capable of orchestrating several biological processes, including the immune response, through specific transporters and receptors [26, 27, 28]. Nevertheless, whether and how lactate regulates the differentiation fate of ICM has not been clearly elucidated. We therefore hypothesised that lactate, the end metabolite of glycolysis, may serve as a key regulator of the second cell fate decision in pre‐implantation embryos, thus bridging the hallmark metabolic remodelling and lineage commitment within ICM.

The orchestrated histone modifications, including H3K27ac and H3K4me3, are essential for normal PrE specification [29, 30]. This is reminiscent of histone lactylation, a recently discovered histone modification that is induced by lactate and enhances chromatin accessibility and activates gene transcription [31, 32]. In contrast to those canonical histone modifications, little is known about the role of histone lactylation in regulating lineage commitment within ICM. The exploration of this question will not only enrich the epigenetic mechanism of PrE differentiation, but also, more importantly, couple the developmental metabolic remodelling with the second cell fate decision in pre‐implantation embryos.

In the present study, using embryonic stem cells (ESCs), in vitro and in vivo embryos as the model, we identify lactate as a signalling molecule and epigenetic substrate that promotes PrE formation via intercellular interaction with paracrine FGF signalling during ICM differentiation, forming a synergistic positive feedback loop that is critical for PrE differentiation. In addition, the FGF‐lactate loop increases histone lactylation levels in blastocysts. Our study demonstrates a model in which self‐produced metabolite and paracrine growth factor synergistically regulate PrE specification, probably via a metabolism‐coupled epigenetic mechanism, highlighting complex multilayer regulation of lactate in lineage commitment during early development.

## 2 Materials & Methods

### 2.1 In Vitro Culture and Collection of Mouse Embryos

Cumulus‐oocyte complexes (COCs) were obtained from8–10‐weeks female mice superovulated with 5 IU PMSG and 5 IU HCG. Then in vitro fertilisation (IVF) was performed, and embryos were cultured in KSOM medium under mineral oil overlay at 37°C in a 5% CO_2_ atmosphere for 72 h. Then, embryos were transferred to DMEM supplemented with 1 mM sodium pyruvate solution, 1:100 dilution of nonessential amino acid solution, and 1:200 dilution of penicillin–streptomycin solution to eliminate potential interference from sodium lactate present in KSOM, followed by an additional 30‐h culture period to obtain late blastocysts. For embryo transfer, the late blastocysts from each group were surgically transplanted into the uterine horn of synchronised pseudopregnant female recipients.

### 2.2 In Vitro Embryo Treatment With Sodium Lactate, Sodium Oxamate or α‐Cyano‐4‐Hydroxycinnamic Acid

All in vitro embryo treatments followed a standardised protocol. Following 72 h pre‐culture in KSOM medium, in vitro embryos were subjected to experimental treatments in DMEM medium containing sodium lactate (10 mM, 20 mM, or 50 mM), sodium oxamate (1 mM, 10 mM, or 50 mM) or α‐cyano‐4‐hydroxycinnamic acid (CHC, 1 mM) for 30 h to assess the regulatory role of lactate or lactate shuttle in PrE specification (Figure S2). Based on initial screening results, the 50 mM concentrations of both sodium lactate and sodium oxamate were selected for subsequent mechanistic investigations.

### 2.3 In Vivo Embryo Treatment With Sodium Oxamate or α‐Cyano‐4‐Hydroxycinnamic Acid

ICR female mice (8 weeks old) were mated with ICR male mice at 20:00. Mice were checked for copulation plugs the next morning, and embryos were considered embryonic day 0.5 (E0.5) on the day of plug detection. At E2.5, pregnant mice received intraperitoneal injections once daily of either sodium oxamate (400 or 600 mg/kg) or CHC (1.5 mg/kg) and these two compounds were administered once daily between 09:00–10:00. Pregnant dams at E4.25 were euthanised by cervical dislocation; late‐stage blastocysts were collected from the uterus by flushing with phosphate‐buffered saline.

### 2.4 Collection of E6.5 Embryos

Pregnant mice at E6.5 were euthanised via cervical dislocation. The complete uterine tract was surgically isolated through meticulous removal of periuterine adipose deposits and connective tissue. Longitudinal hysterotomy along the anti‐mesometrial axis was performed to expose the decidual–myometrial interface. Decidual capsules were then carefully liberated from the inner circular myometrial layer through mechanical separation. Subsequently, embryonic proper was precisely isolated in PBS with the separation of the extra‐embryonic components (including the ectoplacental cone and parietal/visceral yolk sac derivatives). Then, E6.5 embryos were fixed in 4% paraformaldehyde for subsequent analyses.

### 2.5 Cell Culture

ESCs employed in this study were PGK12.1, a gift from Ingolf Bach. ESCs were routinely maintained in KnockOut DMEM supplemented with 10% FBS, 1000 U LIF, nonessential amino acids, 2 mM GlutaMAX, and 550 μM β‐mercaptoethanol on 0.1% gelatin‐coated plates. PGK12.1 suspensions were prepared with densities of 2–5 × 10^^^4 cells when they were differentiated by suspension culture of embryonic bodies (EBs). After two days of culture, EBs were seeded into low‐adherence vessels to continue differentiation.

### 2.6 Western Blotting

100 embryos or cultured cells from one well of a six‐well plate of each group were collected and lysed in RIPA buffer (Beyotime Biotechnology) containing the Protease and Phosphatase Inhibitor Cocktail (Beyotime Biotechnology). About 20 μg protein for cells or protein from 100 blastocysts were separated by 12% SDS‐PAGE and then transferred onto hydrophobic polyvinylidene fluoride (PVDF) membranes (Millipore, Billerica, MA, USA). The membranes were blocked with 5% non‐fat dried milk in Tris‐buffered saline containing 0.1% Tween‐20 (TBST) and then incubated with primary antibodies overnight at 4°C. The next day, horseradish peroxidase (HRP)‐conjugated secondary antibodies were incubated, and protein bands were detected using an ImageQuant LAS 4000 (GE Healthcare, Chicago, IL, USA). The detailed information of primary and secondary antibodies used for western blotting in this study is listed in Table S1.

### 2.7 RNA Extraction and Quantitative Real‐Time PCR (qRT‐qPCR)

Total RNA of 30 embryos or cells from one well of a six‐well plate of each group was extracted using TRIzol (15,596,018, Invitrogen). Reverse transcription was performed with HiScript III RT SuperMix for qPCR (+gDNA wiper) (R323‐01, Vazyme). qRT‐PCR was performed with SsoFast EvaGreen Supermix (172–5201, Bio‐rad) on a LightCycler 96 Real‐time PCR system (CFX96, Bio‐rad). The stability validation of the candidate reference genes was identified with NormFinder software, and *β‐actin* was selected as the reference gene based on its stability in response to the presence or absence of FGF or lactate signalling. The relative expression level of genes was normalised to *β‐actin*. The data were analysed using the 2^−ΔΔCt^ method. The results of stability validation for the candidate reference genes and primers are listed in Table S1.

### 2.8 ESCs Differentiation Into Extra‐Embryonic Endoderm (XEN) Cells

Derivation of XEN cells was performed according to described previously [33, 34]. Briefly, PGK cells were dissociated into single cells with 0.05% Trypsin (Sigma) and seeded at a density of 5000 cells/cm^2^ in standard XEN medium (RPMI 1640 supplemented with 15% FBS, 2 mM GlutaMAX and 0.1 mM β‐mercaptoethanol). Twenty‐four hours after initial plating, the media were changed to fresh cXEN derivation medium (standard XEN medium supplemented with 0.01 μM all‐trans retinoic acid plus 10 ng/mL activin A). Cells were cultured in cXEN derivation medium for 2 days. Then cells were cultured in standard XEN medium again (Figure S5A,B).

### 2.9 Immunofluorescence

For embryos: samples were fixed in 4% paraformaldehyde (PFA) at 4°C overnight and then permeabilised with 0.5% Triton X‐100/0.1% PVA at room temperature for 1 h. The embryos were next blocked in 1% BSA at 4°C for 6 h and then incubated with primary antibodies overnight at 4°C. For adherent cells: cells were fixed in 4% PFA for 20 min at room temperature and then permeabilised with 0.3% Triton X‐100 at room temperature for 20 min. The embryos were next blocked in 1% BSA at room temperature for 1 h and then incubated with primary antibodies overnight at 4°C. The next day, embryos were incubated with Alexa Fluor‐488 and Alexa Fluor‐594 labelled secondary antibodies for 1 h at room temperature. For the embryoid body sections, samples were fixed in 4% PFA at room temperature for 1 h, embedded in O.C.T. compound (Tissue‐Tek) and sectioned at 10 μm using a cryostat. Sections were incubated in permeabilisation solution (0.3% Triton X‐100) for 20 min at RT and then in blocking solution (10% fetal sheep serum, 0.1% Triton X‐100) for 1 h at RT. The incubation with primary antibodies and fluorochrome‐conjugated secondary antibodies was performed in the blocking solution at 4°C overnight and RT for 1 h respectively. Finally, all samples were counterstained with DAPI. The fluorescence signals were imaged using a BX51 microscope (Olympus) or laser scanning confocal microscopy (Al Cell Imaging System; Nikon). The information of primary and secondary antibodies used for immunofluorescence in this study is listed in Table S1.

### 2.10 Measurement of Lactate Levels

The lactate level was measured using the Lactate Assay Kit (MAK064, Sigma Aldrich) according to the manufacturer's instructions. Briefly, embryos or cells were washed by PBS. 100 embryos or 10^6^ cells were collected, lysed, and sonicated on ice and suspended in Lactate Assay Buffer. For all conditions, a total of 50 μL of the cell suspension was utilised in a 96‐well plate. A duplicate standard lactate curve was prepared, encompassing concentrations of 0 (blank), 20, 40, 60, 80, and 100 pmole/well, and Lactate Assay Buffer was added to each well to achieve a final volume of 50 μL. Next, 50 μL of the Master Reaction Mix was added to each well, the contents were thoroughly mixed, and the plate was incubated for 30 min at room temperature. The absorbance was then measured at 570 nm using a Tecan Infinite 200 spectrophotometer (Mannedorf, Switzerland).

### 2.11 Cleavage Under Targets and Tagmentation (CUT&tag)

CUT&Tag analysis was performed with the Hyperactive Universal CUT&Tag Assay Kit for Illumina (TD903‐01, Vazyme Biotech) according to the manufacturer's instructions. In brief, about 50,000 intermediate XEN (iXEN) cells at d4 of differentiation with or without 20 mM sodium lactate treatment were collected, and their nuclei were extracted and then bound by activated concanavalin A‐coated magnetic beads. Specific primary antibody H4K12la (1:50, PTM‐1401, PTM BIO) was used in this study overnight at 4°C. After being incubated with goat anti‐rabbit IgG H&L (ab6702, Abcam), cells were treated with pG‐Tn5 adapter complex and then fragmented by proteinase K. The DNA fragments were amplified and purified for next‐generation sequencing (NGS).

### 2.12 Analyses of CUT&tag Data

The quality assessment of raw data was performed using FastQC (v 0.11.9). Then cleaned reads were aligned to the mm10 genome with Bowtie2 (v 2.4.1) and the obtained Bam files were converted from Sam files using samtools (v1.11). Genome coverage bedGraph files for UCSC genome browser were generated by deeptools (v 3.4.3). Peaks were generated by MACS2 (v 2.2.7.1) callpeak with q < 0.01. Promoters were defined as transcription start site ±3 kb. Differential analysis of CUT&Tag peaks was performed by *R* package DiffBind (v3.4.11) with *p* < 0.05.

### 2.13 Reanalysis of Single‐Cell Transcriptomic Data of ICM, PrE and EPI

The single‐cell transcriptomic data, with a GEO accession number of GSE100597, was downloaded from NCBI and utilised for comprehensive gene expression analysis across EPI, PrE and ICM. Cells from E4.5 embryos were segregated into the EPI and PrE cells by exclusive expression of known markers, such as *Nanog*, *Esrrb*, *Fgf4* (EPI) and *Gata6*, *Pdgfra*, *Gata4*, and *Sox17* (PrE). The “intermediate” cells that express both markers were excluded from subsequent analyses. And the 689 differential expression genes (DEGs) (adjusted *p* value ≤ 0.05, log2 fold change > 2) enriched for the PrE were obtained from a published study [35]. Database for Annotation, Visualisation and Integrated Discovery (DAVID) Bioinformatics Resources (https://davidbioinformatics.nih.gov) was utilised for KEGG pathway enrichment analysis of these DEGs. The interactions between KEGG pathways were analysed using PathwayConnector (https://pathwayconnector.cing‐big.hpcf.cyi.ac.cy). The heatmap showing the expression of glycolytic genes of ICM, EPI and PrE was generated based on their average expression levels (counts per million, CPM). Principal component analysis (PCA) was conducted using *R* version 4.3.3, based on the expression of all genes across ICM, EPI and PrE. Correlation analysis was performed using *R* version 4.3.3 with Pearson correlation, to examine the relationships between the expression of *Ldha*, *Ldhb*, and marker genes specific to PrE and EPI across ICM, EPI, and PrE.

### 2.14 Statistical Analysis

Data were shown as mean ± standard error of the mean (SEM). All statistical analyses were performed with SPSS20.0 (IBM Corp., USA) and the significance of differences was calculated by two‐tail unpaired student's *t*‐test unless stated otherwise. All experiments were repeated at least 3 independent biological replicates. Differences were considered to be statistically significant at the *p* < 0.05 level (*p < 0.05, **p < 0.01, ***p < 0.001).

## 3 Results

### 3.1 Lactate Production Is Tightly Coupled With the Formation of Embryonic Primitive Endoderm

**FIGURE 1:**
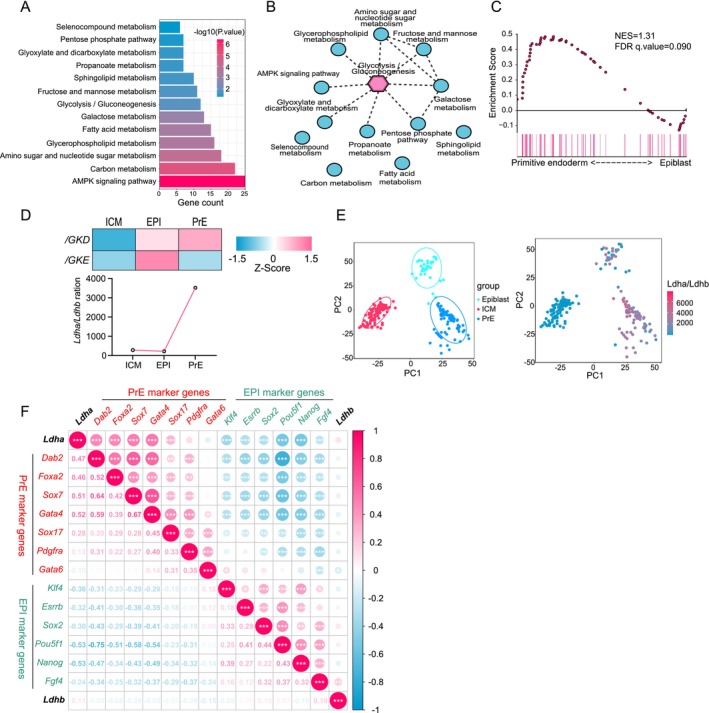
Glycolysis and lactate synthesis is the most active in PrE. (A) KEGG enrichment analysis of differentially highly expressed genes in PrE compared to EPI (GSE100597). (B) Interaction network of KEGG metabolic pathways in (A) analysed by PathwayConnector. (C) The GSEA analysis for genes of glycolysis/gluconeogenesis pathway between PrE and EPI. (D) Analysis of expression of *Ldha*, *Ldhb* and ratio of *Ldha*/*Ldhb* in ICM, EPI and PrE. (E) Analysis of the *Ldha*/*Ldhb* ratio in ICM, EPI and PrE populations by PCA. (F) Pearson correlation heatmap between the expression of *Ldha*, *Ldhb* and marker genes of EPI (green) and PrE (red).

To explore the metabolic changes that characterise ICM differentiation into EPI and PrE, we reanalysed single‐cell transcriptomic data obtained from mouse E3.5 and E4.5 embryos [35]. KEGG pathway enrichment analysis showed that genes exhibiting higher levels in PrE compared to EPI were functionally associated with various metabolic pathways, including glycolysis, fatty acid metabolism, pentose phosphate pathway, etc. (Figure 1A), suggesting that the metabolic differences of ICM cells in blastocysts may be closely related to their differentiation fate. Next, we performed pathway interaction analysis by Pathway Connector [36] to visualise the interactive relationship among these metabolism‐related KEGG pathways. Results suggested that glycolysis/gluconeogenesis occupied a central position in the metabolic differences between PrE cells and EPI cells (Figure 1B). Gene Set Enrichment Analysis (GSEA) further indicated that the glycolysis/gluconeogenesis pathway was the most active in PrE cells, suggesting a key role of glycolysis in driving ICM to differentiate into PrE (Figures 1C and S1A–C).

Induction of glycolysis in PrE was reminiscent of lactate. Thus, we asked if lactate participated in PrE specification. Lactate dehydrogenase A (LDHA) and Lactate dehydrogenase B (LDHB) are key enzymes responsible for the conversion between the glycolysis products, pyruvate and lactate [37]. By reanalysing the single embryo RNA‐sequencing data in our previous study [38] to examine the expression dynamics of *Ldha* and *Ldhb* during pre‐implantation development from zygote to blastocyst stage, we found that the *Ldha*, the enzyme converting pyruvate to lactate, was increasingly upregulated during pre‐implantation development, whereas *Ldhb* showed completely inverse dynamics, leading to a robust increase in the *Ldha*/*Ldhb* expression ratio, a reliable indicator of the synthetic capacity of lactate within cells [39], upon the blastocyst formation and early differentiation (Figure S1D). Furthermore, in line with the high glycolytic activity within PrE cells, PrE cells showed the highest *Ldha*/*Ldhb* expression ratio among different cell populations during differentiation of the blastocysts, suggesting a higher lactate synthesis activity in PrE cells (Figure 1D). UMAP analysis at single‐cell level showed that the PrE population was characterised by the highest *Ldha*/*Ldhb* expression ratio that was distinct from either ICM or EPI populations (Figure 1E). Interestingly, *Ldha* exhibited strong positive correlations with PrE marker genes while being inversely associated with EPI marker genes (Figure 1F). These results indicated that the PrE formation is tightly coupled with *Ldha* upregulation and *Ldhb* downregulation. Therefore, lactate may promote the specification of ICM cells into PrE cells.

### 3.2 Lactate Promotes the Formation of Embryonic Primitive Endoderm and Enhances Post‐Implantation Development

**FIGURE 2:**
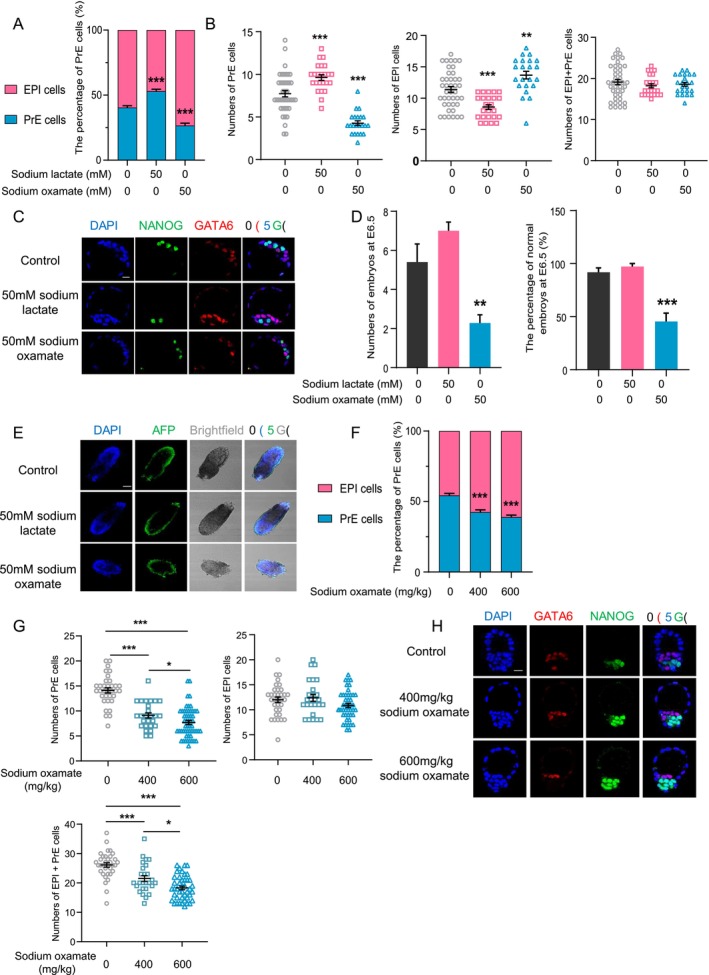
Sodium lactate treatment during blastocyst stage promotes PrE formation and enhances post‐implantation embryonic development. (A, B) Quantitative analysis of the percentage of PrE cells (A) and cell numbers (B) of PrE (GATA6) and EPI (NANOG) in late blastocysts treated with 50 mM sodium lactate or sodium oxamate. (C) Confocal images of late blastocysts immunostained for NANOG (EPI) and GATA6 (PrE), scale bars = 10 μm. (D) The effects of sodium lactate or sodium oxamate treatment during PrE formation on post‐implantation embryonic development. (E) Confocal images of E6.5 embryos immunostained for AFP (VE), scale bars = 100 μm. (F, G) Quantitative analysis of the percentage of PrE cells (F) and cell numbers (G) of PrE and EPI in E4.25 blastocysts in vivo treated with sodium lactate. (H) Confocal images of E4.25 blastocysts in vivo immunostained for NANOG (EPI) and GATA6 (PrE), scale bars = 10 μm. The data represents as mean ± SEM, *p < 0.05, **p < 0.01, ***p < 0.001.

Having confirmed the tight correlation between lactate production and the PrE formation, we next attempted to functionally determine the developmental role of lactate in PrE formation. A recent study demonstrated that lactate and derived histone lactylation are important for zygotic genome activation (ZGA) in 2‐cell embryos [40]. To exclude the possible prolonged effect of ZGA on PrE specification, we transiently treated embryos from the stage (E3.0) before blastocyst formation to late blastocyst (E4.25), covering PrE and EPI specification with sodium lactate or sodium oxamate, the selective inhibitor of LDHA (Figure S2A). Increased cellular lactate content, due to exogenous sodium lactate supplementation (Figure S3A), significantly promoted the PrE specification without affecting the ICM cell number, with increased PrE proportions and enhanced expression of PrE marker genes (Figure 2A–C, S3B), and this effect was dose‐dependent (Figure S3E,F). Conversely, inhibiting lactate synthesis by sodium oxamate (Figure S3A) significantly repressed PrE specification without affecting the ICM cell number, as shown by the decreased PrE cell proportion and increased expression of EPI marker genes (Figures 2A–C and S3C), which was also dose‐dependent (Figure S3G,H). These results indicate that lactate is a key factor fine‐tuning second lineage segregation in the blastocyst by facilitating the PrE formation. Because PrE is the main origin of the visceral endoderm (VE) of the visceral yolk sac and thus is vital for normal embryonic development [41], we next investigated whether promoted or impaired PrE formation via manipulating lactate content would affect the implantation and post‐implantation development. To this end, late blastocysts transiently treated with sodium lactate or sodium oxamate were transferred into pseudopregnant female recipients (Figure S2B). Although exogenous lactate exposure during blastocyst formation did not seem to affect peri‐implantation development, inhibiting endogenous lactate production in blastocysts led to a significant decrease in both implantation success rate and post‐implantation development rate (Figure 2D,E). These results demonstrated that the effects of lactate on PrE specification are crucial for post‐implantation development. The promoting role of lactate in PrE formation was further supported by an in vivo model (Figure 2F–H and Figure S3D), in which sodium oxamate was intraperitoneally injected during the time window from the blastocyst formation to segregation of PrE and EPI (Figure S2C).

### 3.3 Lactate Promotes Extra‐Embryonic Endoderm Differentiation of Mouse ESCs

**FIGURE 3:**
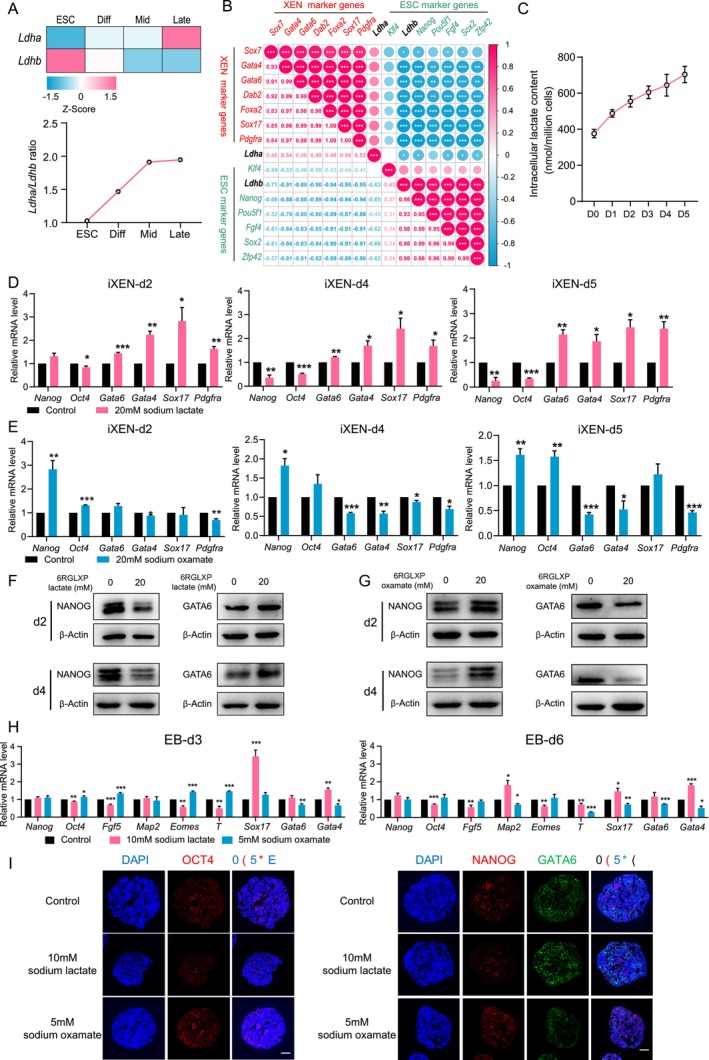
Lactate promotes ESCs differentiation into XEN cells. (A) *Ldha*, *Ldhb* expression and ratio of *Ldha*/*Ldhb* at different stages during ESCs differentiation into XEN cells (GSE77783). Diff: The early stage of differentiation, Mid: The mid stage of differentiation, Late: The late stage of differentiation. (B) Pearson correlation heatmap between the expression of *Ldha*, *Ldhb* and marker genes of ESCs (green) and XEN cells (red) during the differentiation of ESCs to XEN cells. (C) The dynamics of intracellular lactate content during the process of inducing ESCs to differentiate into iXEN cells. (D, E) mRNA expression levels of pluripotent genes and PrE marker genes at different days of differentiation upon sodium lactate (D) and sodium oxamate (E) treatment. (F, G) Protein expression of NANOG and GATA6 at day 2 and day 4 of differentiation upon sodium lactate (F) and sodium oxamate (G) treatment. (H) mRNA expression of marker genes at day 3 and day 6 in EBs treated with sodium lactate or sodium oxamate. (I) Confocal images of EBs at day 6 under different conditions immunostained for OCT4, NANOG and GATA6, scale bars = 100 μm. The data represents as mean ± SEM, *p < 0.05, **p < 0.01, ***p < 0.001.

Inducing differentiation of ESCs into XEN cells is a widely used in vitro model for investigating the mechanism of PrE specification [33, 42], which excludes the possible paracrine effect from trophectoderm in blastocysts. By reanalysing the publicly available dynamic RNA‐seq during ESCs differentiation into XEN cells (GSE77783) [43], we observed that *Ldha*/*Ldhb* expression ratio was also enhanced upon differentiation (Figure 3A), and *Ldha* expression levels were also positively correlated with XEN marker genes while negatively correlated with ESC marker genes (Figure 3B), recapitulating the metabolic changes and PrE commitment in the blastocyst.

These results were further validated using the ESCs differentiation model (Figure S5A,B), in which ESCs were differentiated into XEN cells, named induced XEN (iXEN) cells. Additionally, an elevation in cellular lactate production was observed upon differentiation (Figure 3C). In line with the findings in blastocysts, sodium lactate promoted ESCs differentiation in a dose‐dependent manner (Figures 3D,F and S4A), while sodium oxamate prevented this differentiation (Figures 3E,G and S4B). These results were further confirmed using the EB differentiation model, in which ESCs in high‐lactate conditions preferred to differentiate into XEN cells (Figures 3H,I and S5C‑E). Sodium oxamate treatment resulted in an increased number of OCT4^+^ labelled cells and a decreased number of GATA6^+^ labelled cells (Figure 3I). In summary, using in vivo and in vitro models, we confirmed that lactate promotes the PrE lineage commitment.

### 3.4 Lactate Exerts the Differentiation‐Prompting Effect by Activating FGF‐ERK Signalling

**FIGURE 4:**
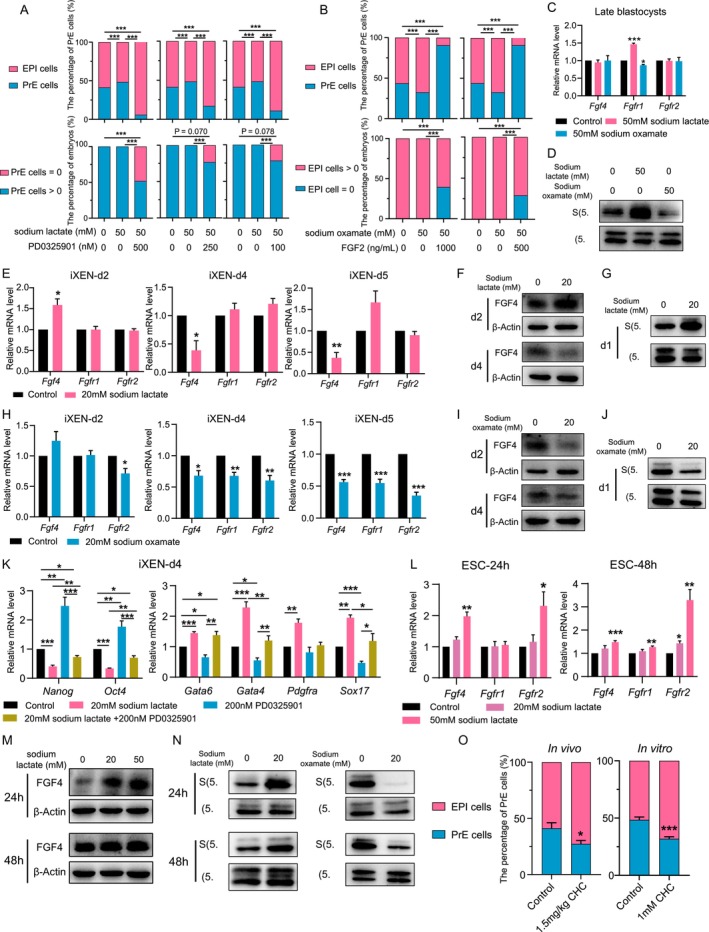
Lactate activates FGF‐ERK signalling in late blastocysts, iXEN cells and ESCs. (A, B) Quantitative analysis of the percentage of PrE cells in late blastocysts (upper) and the percentage of late blastocysts with at least one PrE cell (below). The percentage of PrE cells = number of total PrE cells of all late blastocysts/number of (total PrE cells + total EPI cells) of all late blastocysts in each group. Chi‐square test was used to calculate *p* value. (C) mRNA expression of *Fgf4*, *Fgfr1*, *Fgfr2* in late blastocysts treated with 50 mM sodium lactate or sodium oxamate. (D) The levels of ERK signalling activation in late blastocysts treated with 50 mM sodium lactate or sodium oxamate. (E, H) mRNA expression of *Fgf4*, *Fgfr1*, *Fgfr2* at different days of differentiation upon 20 mM sodium lactate (E) and sodium oxamate (H) treatment. (F, I) Protein expression of FGF4 at day 2 and dady 4 of differentiation upon 20 mM sodium lactate (F) and sodium oxamate (I) treatment. (G, J) The effects of 20 mM sodium lactate (G) and sodium oxamate (J) on ERK signalling activation at day 1 of differentiation. (K) mRNA expression of pluripotent genes and PrE marker genes at day 4 of differentiation with sodium lactate, ERK signalling inhibitor PD0325901 treatment or co‐treatment. (L) mRNA expression levels of *Fgf4*, *Fgfr1*, *Fgfr2* in ESCs treated with sodium lactate for 24 and 48 h. (M) Protein levels of FGF4 in ESCs treated with sodium lactate for 24 and 48 h. (N) The levels of ERK signalling activation in ESCs treated with sodium lactate or sodium oxamate for 24 h and 48 h. (O) The proportion of PrE in E4.25 blastocysts in vivo treated with or without intraperitoneal injection of 1.5 mg/kg CHC, as well as in late blastocysts in vitro treated with or without 1 mM CHC. The data represents as mean ± SEM, *p < 0.05, **p < 0.01, ***p < 0.001.

It has been well established that EPI‐derived FGF4 stimulates PrE specification in a paracrine manner via FGFR1 and FGFR2 on the membranes of PrE cells [5, 44]. Similarly, lactate activates ERK signalling in several cell types of multiple species [45, 46, 47]. Thus, we next asked whether lactate regulates ICM differentiation in a FGF‐ERK‐dependent manner. Even under ERK signalling blockade via PD0325901, 50% of sodium lactate‐treated embryos retained PrE cells (Figure 4A). Conversely, following high‐dose FGF2 administration (1000 ng/mL), some sodium oxamate‐treated embryos still developed EPI‐lineage cells (Figure 4B). These results implied that lactate might functionally potentiate FGF‐ERK signalling. In addition, sodium lactate activated FGF‐ERK signalling in late blastocysts by upregulating *Fgfr1*, the primary FGF receptor that mediates PrE formation, while sodium oxamate remarkably inhibited FGF‐ERK signalling (Figure 4C,D). These results supported the notion that lactate facilitates PrE formation dependent on the activation of FGF‐ERK signalling.

What is more, sodium lactate transiently stimulated the expression of *Fgf4* in iXEN cells, predominant FGF ligand that stimulates PrE formation during the initial stage of differentiation, although followed by an inhibitory effect (Figure 4E,F and S6A). Given that FGF4 is thought to be essential for the exit of ESCs from the pluripotent state [34], and is downregulated upon differentiation (Figure S6C), lactate may promote ESC differentiation into XEN cells by increasing FGF4 levels at the initial stage. Because ERK signalling pathway is also crucial for mediating the differentiation‐prompting effect of FGF4 during the initial phase of differentiation [34, 48], by which ERK activation is critical for ESC exiting from pluripotency and entering XEN differentiation, we next investigated the effect of lactate on ERK activation in iXEN cells at day 1 and found that ERK was notably activated due to sodium lactate treatment (Figure 4G). However, the lactate‐induced ERK activation was undetectable during the later differentiation stage (Figure S6D), probably due to the lactate's repression of *Fgf4* expression during this stage (Figure 4E), or the progressive decline of ERK activation levels during the differentiation process. Conversely, when lactate production was inhibited by sodium oxamate, *Fgfr2* expression was inhibited in iXEN cells at day 2 and *Fgf4*, *Fgfr*1, and *Fgfr2* were inhibited at day 4–5 of differentiation (Figure 4H,I and S6B), along with the inhibited ERK phosphorylation at day1(Figure 4J). Treatment with an ERK inhibitor diminished the differentiation‐prompting effect of lactate, as revealed by increased expression of pluripotent genes and decreased expression of XEN marker genes in iXEN cells at day 4 (Figure 4K). Besides, the stimulatory effects of lactate on FGF4 expression and ERK signalling were also confirmed using sodium lactate and sodium oxamate in the pluripotent ESCs (Figure 4L,N), suggesting that lactate promotes FGF4 expression in ICM cells. We therefore speculated that lactate from PrE cells enhanced *Fgf4* expression in surrounding EPI cells through the lactate shuttle, which supports the cell–cell delivery of lactate as a signalling molecule. To test this, in vitro cultured embryos were treated with 1 mM α‐cyano‐4‐hydroxycinnamic acid (CHC), an inhibitor for monocarboxylate transporters (MCTs) that are responsible for the lactate shuttle, to impede the lactate shuttle between EPI and PrE cells during ICM differentiation of in vitro embryos (Figure S2A). Alternatively, pregnant mice were intraperitoneally injected with 1.5 mg/kg CHC to repress the lactate shuttle within in vivo embryos (Figure S2C). Blocking the lactate shuttle significantly repressed PrE formation in late blastocysts both in vivo and in vitro (Figure 4O), indicating a critical role of the lactate shuttle in PrE formation. Collectively, our in vitro and in vivo results demonstrated that lactate activated FGF‐ERK signalling, via the lactate shuttle to facilitate PrE formation both in embryos and ESCs.

### 3.5 FGF Signalling May Increase Lactate Production by Repressing Ldhb Expression

**FIGURE 5:**
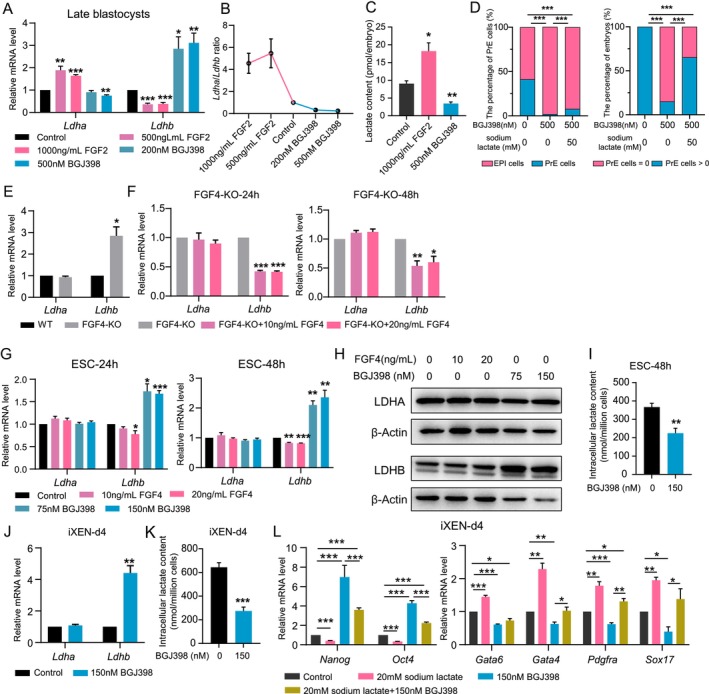
FGF signalling decreased LDHB expression in embryos and ESCs. (A, B) mRNA expression levels of *Ldha* and *Ldhb* (A) and ratio of *Ldha*/*Ldhb* (B) in late blastocysts treated with FGF2 or BGJ398. (C) Lactate contents in late blastocysts treated with FGF2 or BGJ398. (D) Quantitative analysis of the percentage of PrE cells in late blastocysts (upper) and the percentage of late blastocysts with at least one PrE cell (below) treated with BGJ398 or co‐treated with BGJ398 and sodium Lactate. Chi‐square test was used for statistical analysis. (E) mRNA expression levels of *Ldha* and *Ldhb* in FGF4‐KO ESCs. (F) mRNA expression levels of *Ldha* and *Ldhb* in FGF4‐KO ESCs treated with FGF4 for 24 or 48 h. (G, H) mRNA (G) and protein expression (H) of LDHA and LDHB in ESCs treated with FGF4 or BGJ398 for 24 or 48 h. (I) Intracellular lactate content in ESCs treated with or without 150 Nm BGJ398 for 48 h. (J) mRNA expression levels of *Ldha* and *Ldhb* in iXEN cells at day4 treated with BGJ398. (K) Intracellular lactate content in iXEN cells at day 4 treated with or without 150 Nm BGJ398. (L) mRNA expression levels of pluripotent genes and PrE marker genes in iXEN at day 4 with sodium lactate, BGJ398 treatment or co‐treatment. The data represents as mean ± SEM, *p < 0.05, **p < 0.01, ***p < 0.001.

It has been proven that FGF signalling promotes glycolysis and lactate production in cumulus cells and cancer cells, etc. [49, 50, 51]. We asked whether FGF signalling regulates lactate production during the differentiation of ICM to PrE. Because FGF4 acts in a heparin‐dependent manner, FGF2 was frequently used to activate FGFR‐ERK in embryos due to its heparin‐independent action [9, 12, 44], thus we used FGF2 to investigate the role of FGF signalling in lactate production in late blastocysts. Results showed that lactate production was elevated by exogenous FGF2 in late blastocysts, along with the upregulated *Ldha*, ratio of *Ldha*/*Ldhb* and downregulated *Ldhb*, while selective FGFR inhibitor BGJ398 inhibited lactate production (Figure 5A–C). In addition, exogenous sodium lactate alleviated the inhibitory effect of FGFR blockage on PrE formation (Figure 5D).

We then examined the effects of FGF signalling on the expression of *Ldha* and *Ldhb* in ESCs. We first detected *Ldha* and *Ldhb* expression in *Fgf4*‐knockout ESCs [52]. Expectedly, *Ldhb* was significantly increased in *Fgf4*‐knockout ESCs, whereas *Ldha* expression remained unchanged in these cells (Figure 5E). Additionally, exogenous FGF4 and 10 ng/mL heparin supplementation reduced *Ldhb* expression but also did not alter *Ldha* expression in *Fgf4*‐knockout ESCs (Figure 5F). To further confirm the effects of FGF4 on *Ldha* and *Ldhb* expression, wild type ESCs cells were treated with FGF4 supplemented with heparin or BGJ398. The expression of *Ldhb* in ESCs was significantly inhibited by FGF4 and increased by BGJ398, but the *Ldha* expression did not respond to FGF signalling (Figure 5G,H). We noticed that FGF4's repression on *Ldhb* expression was slight, albeit significant, in ESCs, probably due to endogenous high FGF4 level in the pluripotent ESCs [53]. Importantly, BGJ398 treatment suppressed lactate synthesis in ESCs (Figure 5I). Thus, FGF4 may promote lactate production by repressing *Ldhb*.

To further validate the effects of FGF4 on lactate synthesis, we next investigated the effects of FGFR suppression on *Ldha* and *Ldhb* expression and lactate production in iXEN cells at day 4. The results showed that FGFR inhibition dramatically upregulated *Ldhb* but did not affect *Ldha* expression and impeded lactate production in iXEN cells (Figure 5J,K). In addition, FGFR inhibition‐blocked ESC differentiation and XEN differentiation was reversed by sodium lactate, as revealed by changes in pluripotent genes and PrE marker genes (Figure 5L). Thus, these results suggest that FGF signalling stimulates lactate production and PrE formation by inhibiting *Ldhb* expression.

### 3.6 FGF‐Lactate Loop Increases Histone Lactylation Levels in Blastocysts and H4K12la Enrichment at the Promoter Regions of Genes Important for PrE Differentiation

**FIGURE 6:**
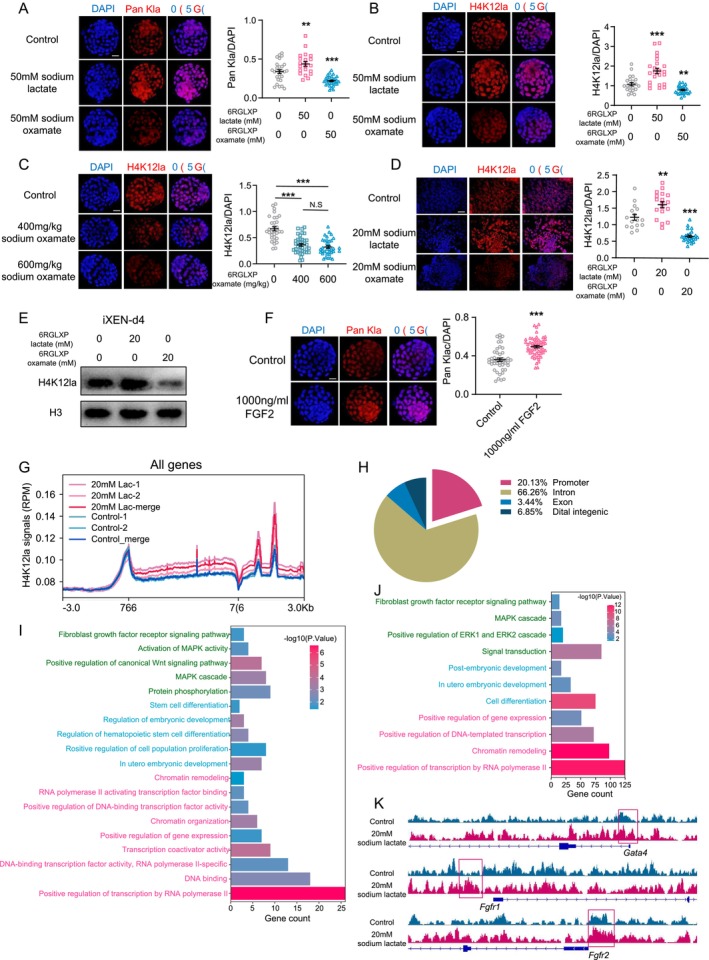
Lactate increased histone lactylation in embryos and iXEN cells. (A, B) PanKla (A) and H4K12la levels (B) in late blastocysts in vitro treated with 50 mM sodium lactate or sodium oxamate, scale bars = 10 μm. The results of quantitative analysis were presented in the right panel. (C) The effects of intraperitoneal injection with sodium oxamate on H4K12la levels in E4.25 blastocysts in vivo. (D, E) H4K12la levels in iXEN cells at day 4 treated with 20 mM sodium lactate or sodium oxamate, measured by immunofluorescence (D) and western blot (E), scale bars = 50 μm. (F) The effects of FGF2 on PanKla levels in late blastocysts. (G) H4K12la CUT&Tag profiles around the gene body of all genes in Control and sodium lactate‐treated iXEN cells at day 4. TSS, transcription start site. TES, transcription end site. (H) Genomic localisation of the 3195 differential H4K12la peaks with lactate treatment. (I, J) Gene ontology terms of the genes with differential H4K12la peaks in promoters (I) or introns (J). (K) Selected genome views for H4K12la CUT&Tag data in iXEN cells at day 4 with or without 20 mM sodium lactate treatment. Genome views: *Gata4* (chr14: 63,849,357–63,872,717), *Fgfr1* (chr8: 26,623,272–26,647,853), *Fgfr2* (chr7: 137,403,028–137,417,257). Genome views for the same genes are on the same vertical scale. The data represents as mean ± SEM, *p < 0.05, **p < 0.01, ***p < 0.001.

Lactate‐derived histone lactylation is a newly identified epigenetic modification that stimulates gene transcription [32] and involves cell fate determination of pluripotent stem cells [54]. The increased lactate production during ICM differentiation into PrE made us wonder whether lactate regulates histone lactylation during PrE differentiation. The results showed that both total histone lactylation levels and H4K12la levels were sensitive to either exogenous lactate supplementation or endogenous lactate synthesis inhibition (Figure 6A,B). This finding was further confirmed using in vivo intraperitoneal injection of sodium oxamate (Figure 6C). In agreement with our results in embryos, H4K12la level also sensitively responded to either exogenous lactate supplementation or endogenous lactate synthesis inhibition in iXEN cells at day 4 (Figure 6D,E). Moreover, in line with the prompting effect on lactate production, exogenous FGF2 also increased histone lactylation levels in late blastocysts (Figure 6F), suggesting the coupled relationship between FGF and lactate signalling.

In order to explore the potential functional mechanism of H4K12la in lactate‐induced PrE formation, we performed CUT&Tag assays for H4K12la in iXEN cells at day 4, treated with or without sodium lactate. The results revealed that lactate supplementation appeared to enhance the enrichment of H4K12la at gene body and 3′ regions (Figure 6G). More detailed analysis showed H4K12la enrichment at 3195 sites was significantly enhanced due to lactate treatment (*p* < 0.05, Table S2). Among these, the majority (66.26%) were located in intron within gene body regions, 20.13% mapped to promoter regions (Figure 6H), and we focused on the genes enriched in these two clusters. Gene ontology (GO) analysis indicated that these genes were enriched in multiple basic processes, such as regulation of transcription and chromatin status. Of note, genes in the canonical pathway that regulate PrE specification were also enriched with H4K12la, suggesting a basal function of lactate‐derived lactylation in PrE specification (Figure 6I). In addition, because histone lactylation has been reported to influence transcription elongation [55], we next analysed the increased lactylation enrichment within intron regions, and also found that histone lactylation may regulate PrE specification via the processes of transcriptional regulation, cell differentiation, and crucial pathways for PrE specification (Figure 6J). More importantly, we found that the H4K12la signals around the transcription start sites of *Gata4*, *Fgfr1*, and *Fgfr2* in iXEN cells at day 4 were enhanced upon sodium lactate treatment (Figure 6K). This result meant that histone lactylation may participate in the PrE specification mainly by promoting the expression of *Gata4*, *Fgfr1*, *Fgfr2*, or other relevant genes.

## 4 Discussion

It has been reported that blastocysts increase their glycolytic activity and decrease oxidative phosphorylation upon ICM differentiation into EPI and PrE [14, 16]. Our in‐depth analysis, intriguingly, revealed that glycolysis and lactate production are more preferentially upregulated in PrE compared to EPI, suggesting that glycolysis and its metabolites contribute to ICM conversion into PrE. Using multiple in vitro and in vivo models, we demonstrate that lactate, the end product of glycolysis, is critical to the differentiation of ICM into PrE, providing novel insights into the functions of lactate in embryonic development.

Multiple lines of evidence underscore the crucial role of the FGF4‐ERK signalling pathway in the formation of PrE [9, 12, 44]. Furthermore, the regulatory relationship between lactate and FGF or ERK signalling has been established in somatic and cancer cells [51, 56, 57, 58, 59]. To the best of our acknowledge, our study is the first to identify the cross‐talk regulation between lactate and FGF signalling in the cell fate determination during early development. Lactate activates FGF‐ERK signalling in late blastocysts, ESCs and iXEN cells. Reciprocally, FGF signalling suppresses *Ldhb* in both late blastocysts, ESCs and iXEN cells, which is in line with a previous study that FGF signalling downregulated *Ldhb* expression in MEF cells [58].

Lactate shuttle from hypoxic cells to oxidative cells is a common phenomenon in tissues exhibiting the Warburg effect, such as cancer and decidua [60, 61, 62]. In these tissues, a large amount of lactate synthesised in hypoxic cells is transported to oxidative cells, where lactate is oxidised back to pyruvate and utilised for the tricarboxylic acid cycle to fulfil the energy demands of cell generation. Given the unique position of PrE and EPI, and the fact that glycolysis and lactate production are more active in PrE compared to EPI, it is reasonable that lactate produced in PrE cells may be transferred into EPI cells. Although it is challenging to detect the lactate shuttle between PrE and EPI cells, we demonstrate that the lactate shuttle is important for PrE formation.

Recent studies have revealed that lactate‐derived lactylation of histone lysine residues serves as an epigenetic modification, directly stimulating gene transcription from chromatin [32, 54, 63]. Our current research found that lactate elevated the levels of histone lactylation in late blastocysts and iXEN cells. Specifically, increased H4K12la levels may be involved in chromatin remodelling, gene transcription, and stem cell differentiation. Notably, lactate enhances H4K12la levels at the gene body regions and promoters of genes that may participate in transcription regulation by Polymerase II, because it has been reported that transcription elongation is governed by RNA polymerase II and specific histone modifications, such as H3K18la, H3K36me3 that bind to TSS and gene bodies [55, 64].

**FIGURE 7:**
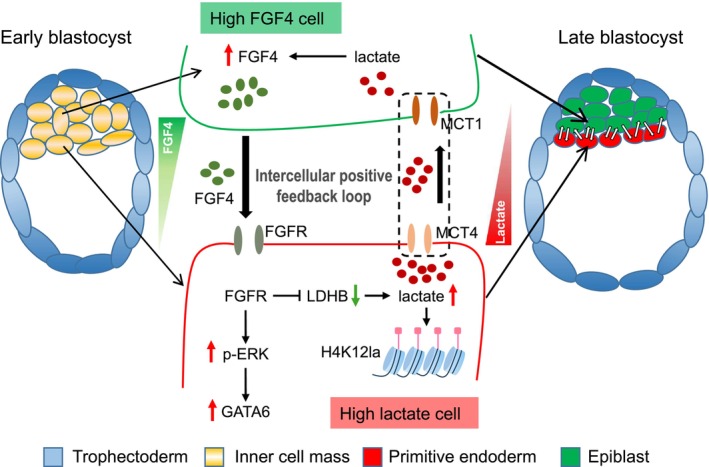
Working model for lactate to promote PrE formation. During the development of early blastocyst to late blastocyst, FGF4 exhibits a bimodal expression pattern in ICM cells, which possess bipotential differentiation capabilities. High FGF4 cells tend to develop into EPI cells, while high‐lactate cells differentiate into PrE cells. In high‐lactate cells, paracrine FGF4 signalling stimulates lactate production and ERK signalling through FGFR. Then lactate and activated ERK signalling enhance the expression of PrE marker genes. Lactate can also be transported to adjacent cells with high FGF4 levels, where it activates FGF4 expression, thereby facilitating the specification program of the PrE. Eventually, lactate promotes PrE lineage specification through an intercellular positive feedback loop that couples paracrine FGF signalling.

We therefore propose a model of intercellular positive feedback loop in which paracrine FGF4 from EPI precursor cells promotes lactate synthesis within PrE precursor cells, while the increased lactate in turn stimulates FGF‐ERK signalling via lactate shuttling (Figure 7). Additionally, the increased lactate in PrE precursor cells also directly promotes PrE specification by elevating the level of histone lactylation, bridging the epigenetic reprogramming and metabolic remodelling during the second cell fate determination.

## Author Contributions

Xiao Hu designed the study, performed experiments, analysed the data, performed bioinformatics analysis and wrote the manuscript; Yawen Tang performed experiments, analysed the data; Wei Zhao and Juan Liu performed experiments, analysed the data and wrote the manuscript; Zhize Liu performed experiments; Qianyin Yang and Jianhui Tian contributed to the discussion; Lei An and Shumin Wang supervised the study and contributed to writing.

## Ethics Statement

All experiments involving laboratory animals were carried out in accordance with the Guide for the Care and Use of Agricultural Animals in Agricultural Research and Teaching and approved by the Institutional Animal Care and Use Committee at the China Agricultural University (approval number: AW70803202‐1‐1).

## Conflicts of Interest

The authors declare no conflicts of interest.

## Supporting information


**Figure S1.** Glycolysis activity and *Ldha* expression are upregulated in primitive endoderm. (A) The GSEA analysis for genes of the glycolysis/gluconeogenesis pathway, comparing PrE versus ICM and EPI versus ICM. (B) The expression of important glycolytic enzymes in ICM, PrE and EPI. (C) The expression changes of important glycolytic enzymes in ICM, PrE and EPI. The first arrow shows the comparison of EPI with ICM, the second arrow shows the comparison of PrE with ICM and the third arrow shows the comparison of PrE with EPI. Red arrows indicate increased expression, blue arrows indicate decreased expression. (D) The dynamic of *Ldha*, *Ldhb* expression and *Ldha*/*Ldhb* expression ratio during the development of pre‐implantation embryos.
**Figure S2.** The procedures of treating embryos with sodium lactate, sodium oxamate and CHC. (A) Schematic depiction of treating embryos with sodium lactate, sodium oxamate or CHC. (B) Schematic depiction of embryonic transplantation of late blastocysts treated with sodium lactate or sodium oxamate. (C) Schematic depiction of intraperitoneal injection of sodium oxamate or CHC solution in pregnant female mice.
**Figure S3.** The effects of sodium lactate and sodium oxamate on the expression of marker genes and PrE formation in late blastocysts. (A) Lactate content in late blastocysts treated with 50 mM sodium lactate or sodium oxamate. (B‑D) The effects of sodium lactate and sodium oxamate on *Nanog*, *Sox17* and *Gata6* expression. (E, F) The effects of sodium lactate on the percentage of PrE cells (E) and numbers of PrE cells, EPI cells and EPI + PrE cells in late blastocysts (F). (G, H) The effects of sodium oxamate on the percentage of PrE cells (G) and numbers of PrE cells, EPI cells and EPI + PrE cells in late blastocysts (H). The data represents as mean ± SEM, * *p* < 0.05, ** *p* < 0.01, *** *p* < 0.001.
**Figure S4.** The effects of sodium lactate and sodium oxamate on ESCs differentiation. (A, B) mRNA expression of pluripotent genes and PrE marker genes at different days in iXEN cells treated with sodium lactate (A) and sodium oxamate (B). The data represents as mean ± SEM, * *p* < 0.05, ** *p* < 0.01, *** *p* < 0.001.
**Figure S5.** Differentiation of ESCs into induced XEN cells or embryonic bodies (EBs). (A) The process of ESCs differentiation into induced XEN (iXEN) cells. (B) mRNA expression dynamics of *Nanog* and *Gata6* during ESCS differentiation into iXEN cells. (C) The process of the embryoid body differentiation of ESCs. (D) The images of EBs at day 3 and day 6 in different conditions. scale bars = 400 μm. (E) mRNA expression dynamics of pluripotent genes and marker genes of XEN cells during EB differentiation. The expressions of genes at day 0 were set as one. The data represents as mean ± SEM, * *p* < 0.05, ** *p* < 0.01, *** *p* < 0.001.
**Figure S6.** The effects of sodium lactate and sodium oxamate on *Fgf4*, *Fgfr1* and *Fgfr2* expression in iXEN cells. (A, B) mRNA expression of *Fgf4*, *Fgfr1* and *Fgfr2* at different days in iXEN cells treated with sodium lactate (A) and sodium oxamate (B). (C) mRNA expression dynamics of *Fgf4* during ESCs differentiation. (D) The levels of ERK signalling activation in iXEN cells at day 2 and day 4, treated with 20 mM sodium lactate or sodium oxamate. The data represents as mean ± SEM, * *p* < 0.05, ** *p* < 0.01, *** *p* < 0.001.

**Table S1.** List of primary antibodies and primers used in this study.

**Table S2.** CUT&Tag peaks with increased H4K12la signals in sodium lactate‐treated iXEN cells at d4 compared to control.

## Data Availability

The authors declare that all data supporting the findings of this study are available within the article or are available from the corresponding author upon request.
