# Supplementary figures and images for: Lactate Promotes the Second Cell Fate Decision in Blastocysts by Prompting Primitive Endoderm Formation Through an Intercellular Positive Feedback Loop That Couples Paracrine FGF Signalling
Source: Cell Prolif. 2025 Jun 27;58(10):e70088. doi: 10.1111/cpr.70088 (PMC12508697; doi:10.1111/cpr.70088)

Fig.S1

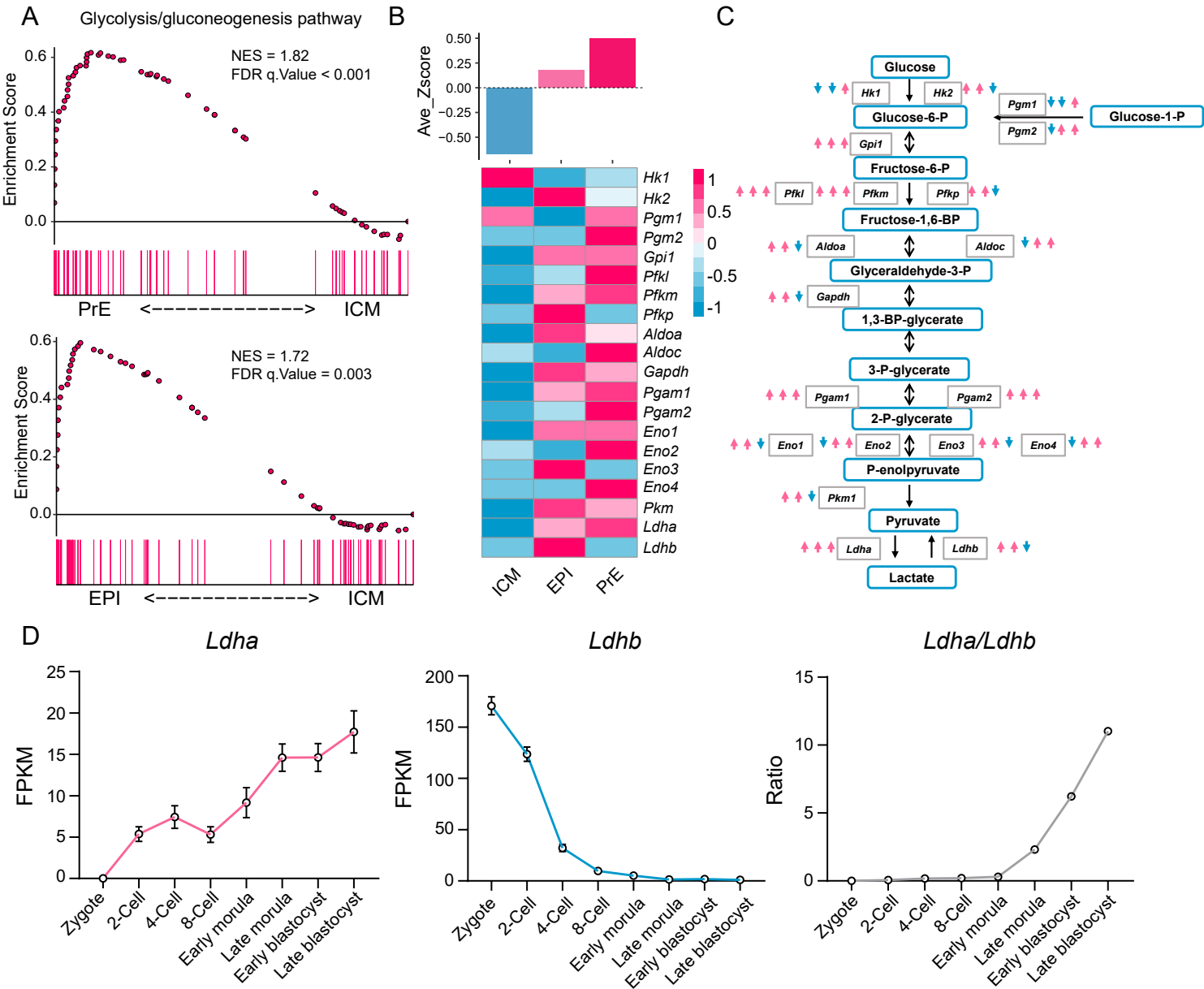

Fig.S2

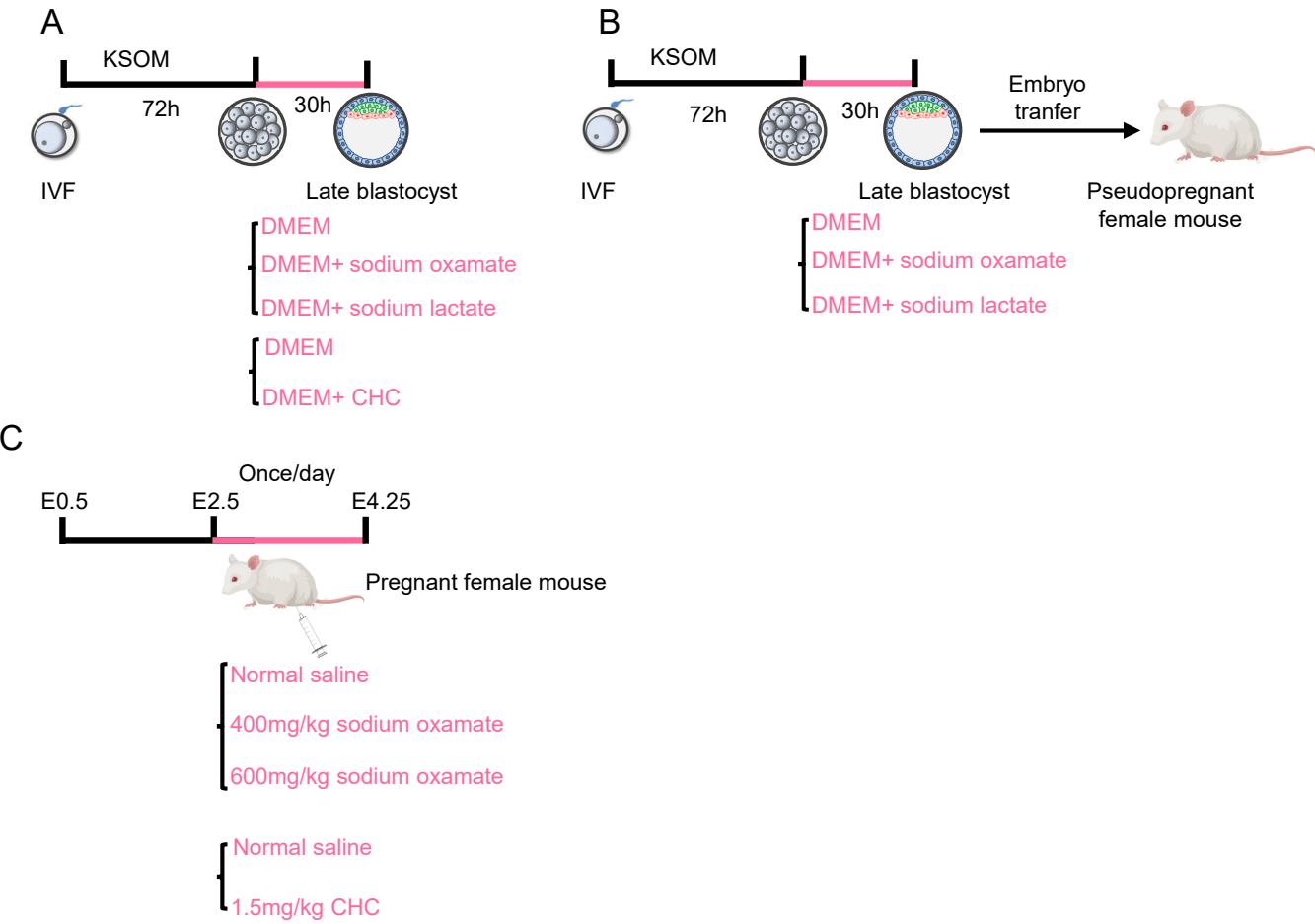

Fig.S3

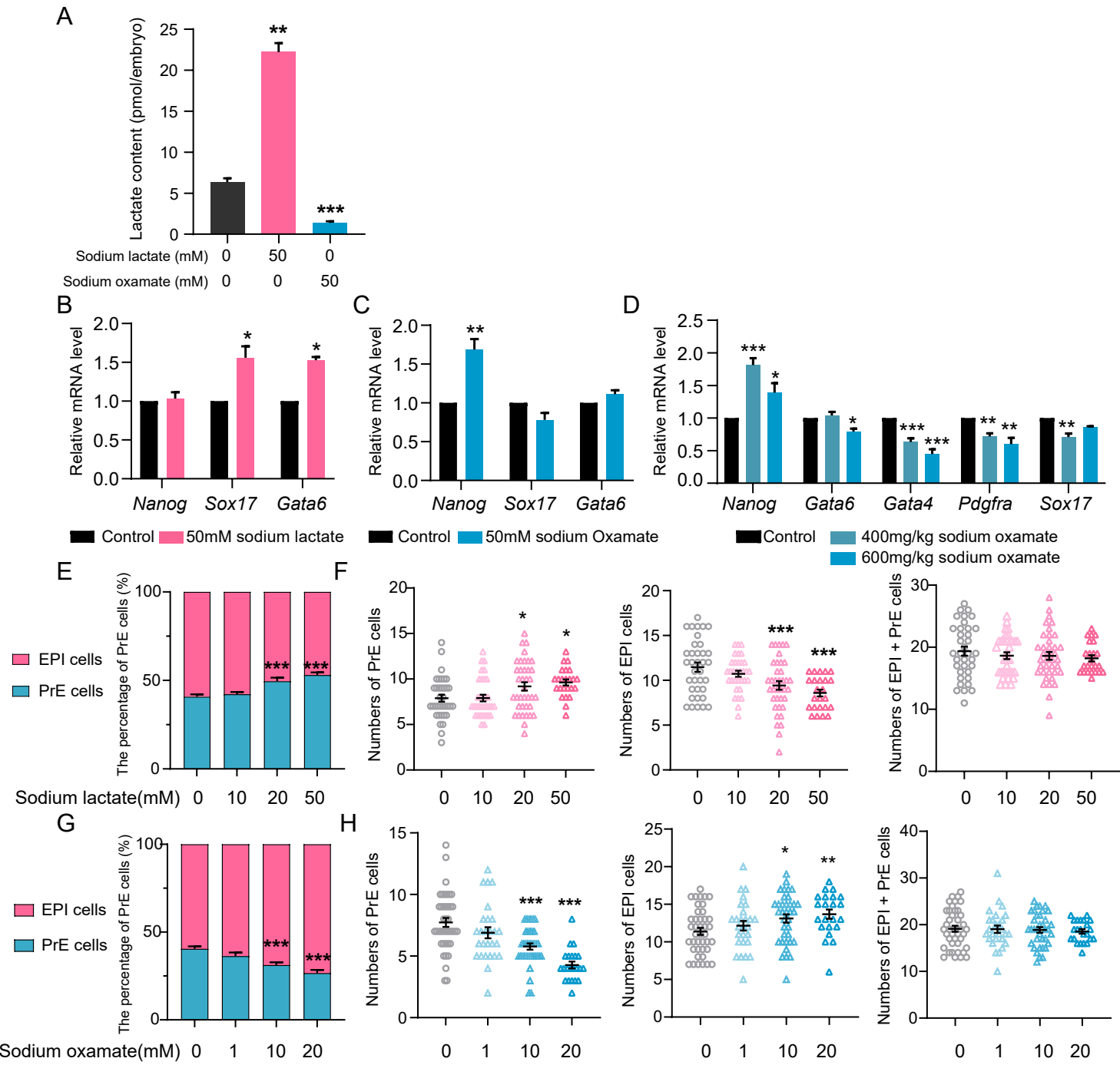

Fig.S4

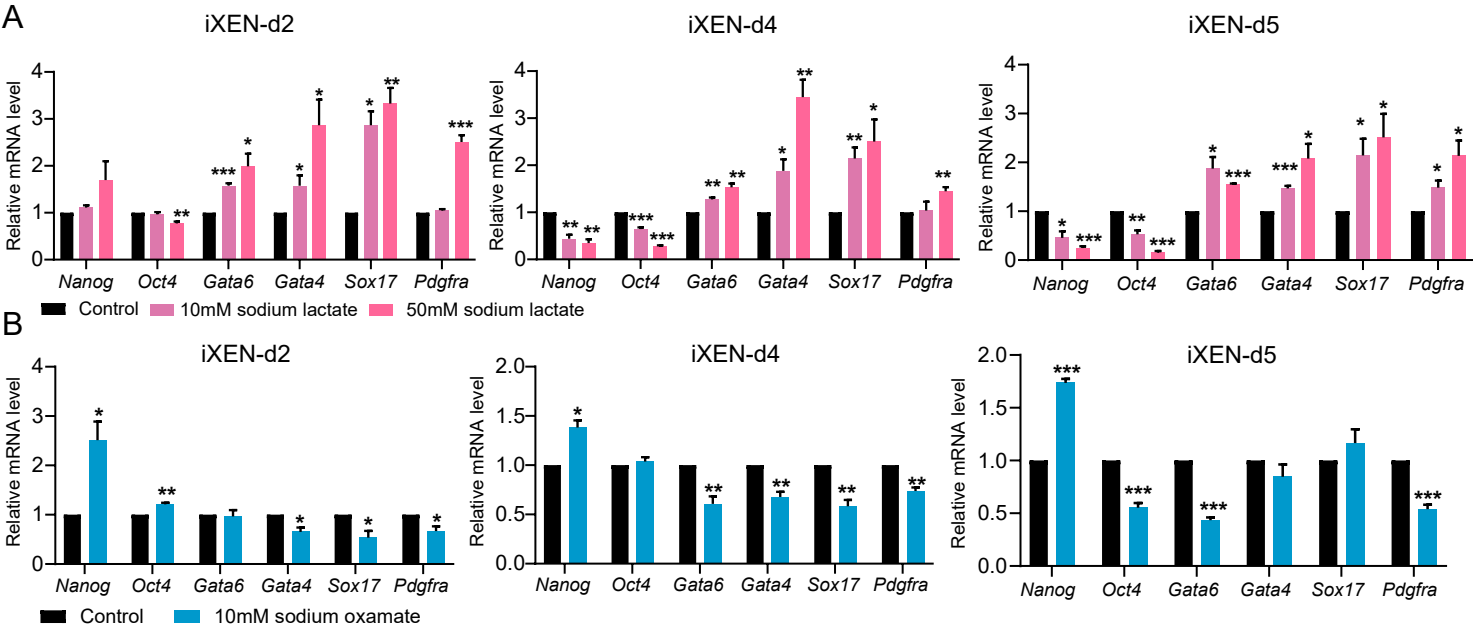

Fig.S5

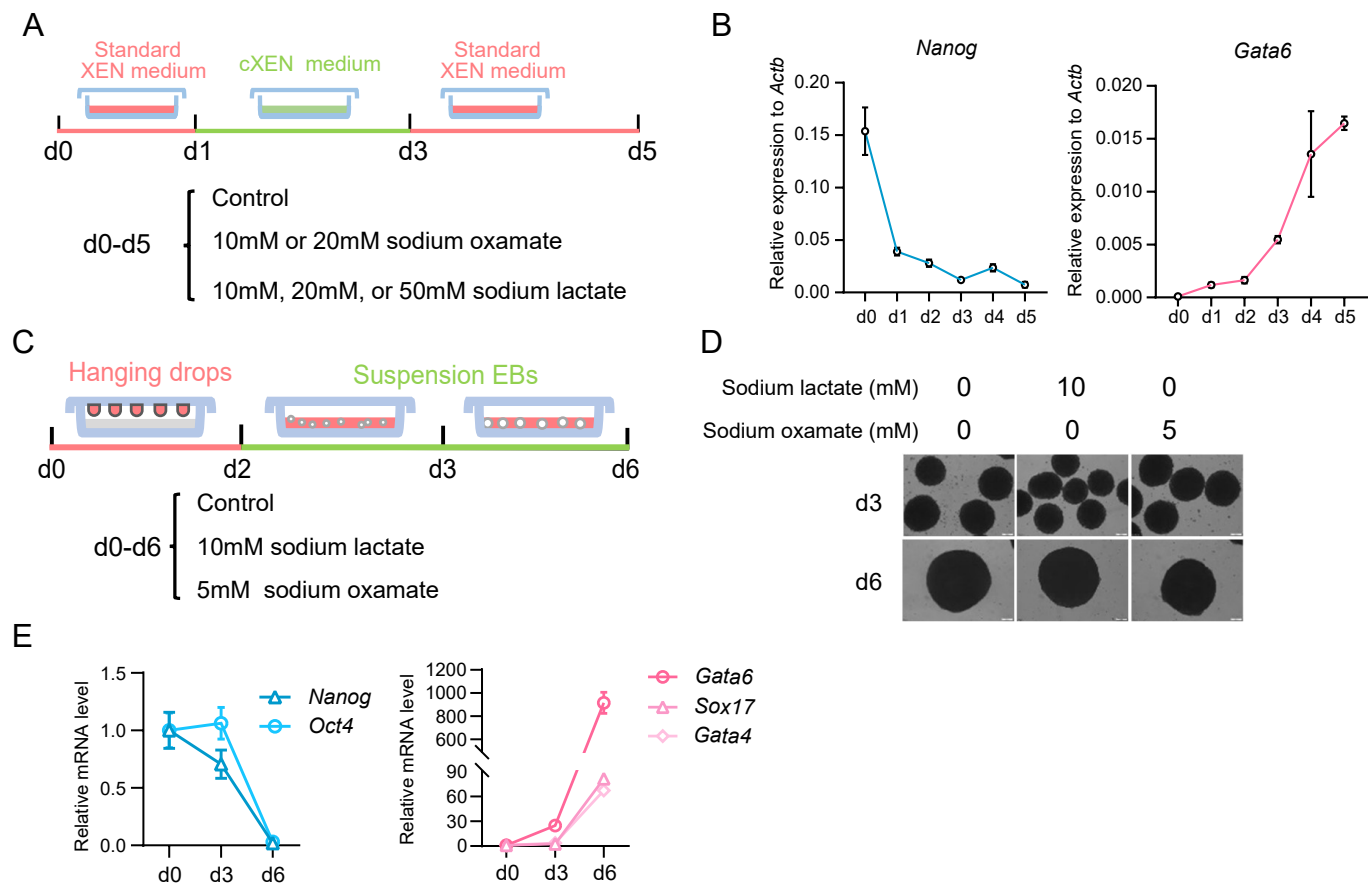

Fig.S6

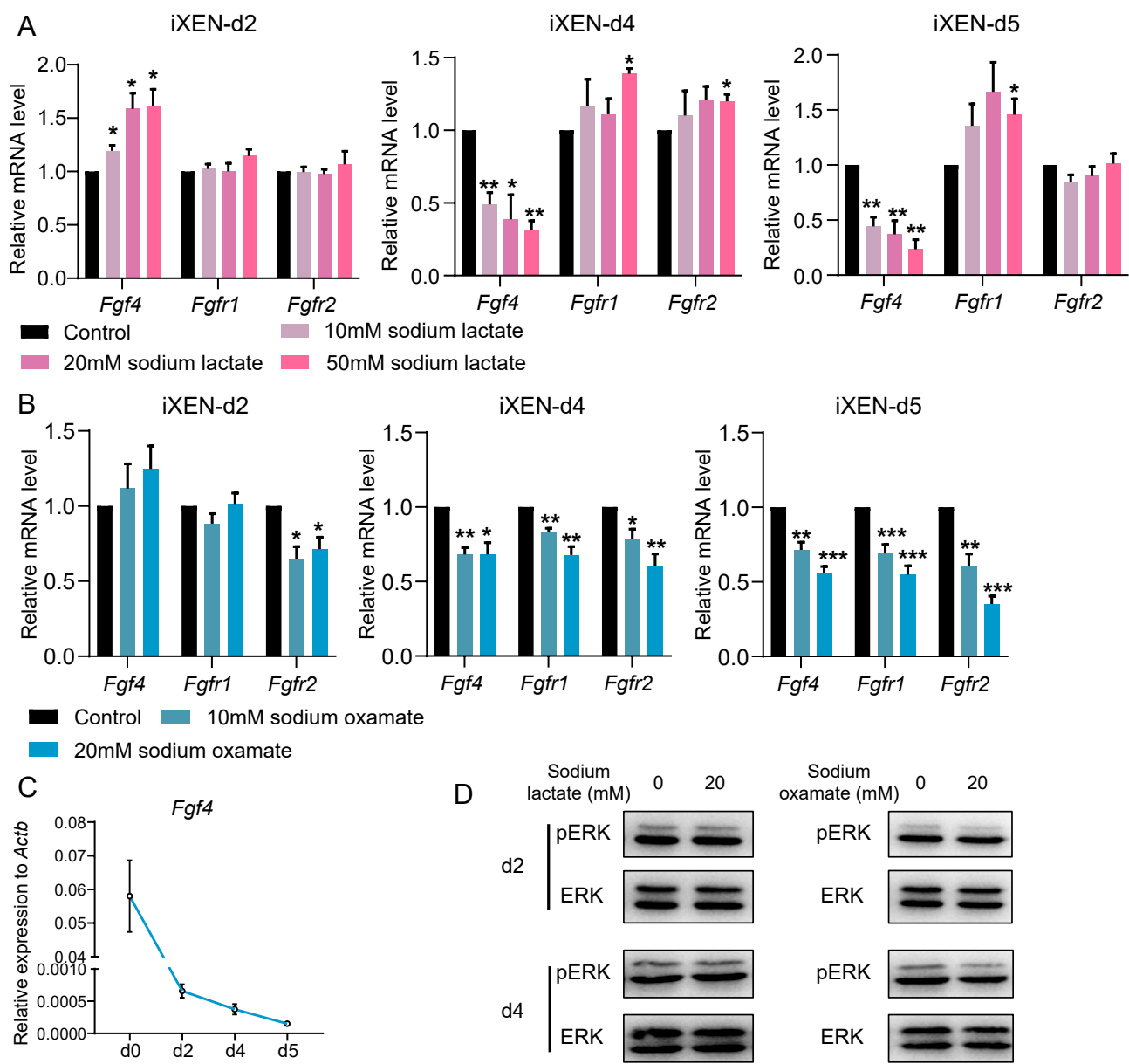

Supplement: Supplementary file 1 — Figure S1. Glycolysis activity and Ldha expression are upregulated in primitive endoderm. (A) The GSEA analysis for genes of the glycolysis/gluconeogenesis pathway, comparing PrE versus ICM and EPI versus ICM. (B) The expression of important glycolytic enzymes in ICM, PrE and EPI. (C) The expression changes of important glycolytic enzymes in ICM, PrE and EPI. The first arrow shows the comparison of EPI with ICM, the second arrow shows the comparison of PrE with ICM and the third arrow shows the comparison of PrE with EPI. Red arrows indicate increased expression, blue arrows indicate decreased expression. (D) The dynamic of Ldha, Ldhb expression and Ldha/Ldhb expression ratio during the development of pre‐implantation embryos. Figure S2. The procedures of treating embryos with sodium lactate, sodium oxamate and CHC. (A) Schematic depiction of treating embryos with sodium lactate, sodium oxamate or CHC. (B) Schematic depiction of embryonic transplantation of late blastocysts treated with sodium lactate or sodium oxamate. (C) Schematic depiction of intraperitoneal injection of sodium oxamate or CHC solution in pregnant female mice. Figure S3. The effects of sodium lactate and sodium oxamate on the expression of marker genes and PrE formation in late blastocysts. (A) Lactate content in late blastocysts treated with 50 mM sodium lactate or sodium oxamate. (B‑D) The effects of sodium lactate and sodium oxamate on Nanog, Sox17 and Gata6 expression. (E, F) The effects of sodium lactate on the percentage of PrE cells (E) and numbers of PrE cells, EPI cells and EPI + PrE cells in late blastocysts (F). (G, H) The effects of sodium oxamate on the percentage of PrE cells (G) and numbers of PrE cells, EPI cells and EPI + PrE cells in late blastocysts (H). The data represents as mean ± SEM, * p < 0.05, ** p < 0.01, *** p < 0.001. Figure S4. The effects of sodium lactate and sodium oxamate on ESCs differentiation. (A, B) mRNA expression of pluripotent genes and PrE marker genes at [file CPR-58-e70088-s002.pdf]
